# Neural Manifestations of Implicit Self-Esteem: An ERP Study

**DOI:** 10.1371/journal.pone.0101837

**Published:** 2014-07-09

**Authors:** Lili Wu, Huajian Cai, Ruolei Gu, Yu L. L. Luo, Jianxin Zhang, Jing Yang, Yuanyuan Shi, Lei Ding

**Affiliations:** 1 Key Laboratory of Mental Health, Institute of Psychology, Chinese Academy of Sciences, Beijing, China; 2 Key Laboratory of Behavioral Science, Institute of Psychology, Chinese Academy of Sciences, Beijing, China; 3 School of Electrical and Computer Engineering, University of Oklahoma, Norman, Oklahoma, United States of America; Vanderbilt University, United States of America

## Abstract

Behavioral research has established that humans implicitly tend to hold a positive view toward themselves. In this study, we employed the event-related potential (ERP) technique to explore neural manifestations of positive implicit self-esteem using the Go/Nogo association task (GNAT). Participants generated a response (Go) or withheld a response (Nogo) to *self* or *others* words and *good* or *bad* attributes. Behavioral data showed that participants responded faster to the *self* paired with *good* than the *self* paired with *bad*, whereas the opposite proved true for *others*, reflecting the positive nature of implicit self-esteem. ERP results showed an augmented N200 over the frontal areas in Nogo responses relative to Go responses. Moreover, the positive implicit self-positivity bias delayed the onset time of the N200 wave difference between Nogo and Go trials, suggesting that positive implicit self-esteem is manifested on neural activity about 270 ms after the presentation of self-relevant stimuli. These findings provide neural evidence for the positivity and automaticity of implicit self-esteem.

## Introduction

Since Greenwald and Banaji's influential paper [1] was published, implicit social cognition has been extensively studied at the behavioral level. In recent years, burgeoning interest has attracted study of the underlying mechanisms of implicit social cognition through the help of cognitive neuroscience methods [2]. To date, research from a cognitive-neural perspective has addressed racial bias [3]–[8], prejudice [9], [10], political attitudes [11], and many other aspects. Surprisingly, while an important area of implicit social cognition, implicit self-esteem has received little attention among cognitive-neural scientists. In our research, we aimed to study neural manifestations of implicit self-esteem.

Implicit self-esteem reflects a kind of automatic, unconscious, and habitual self-evaluation and is often manifested as positive self-associations [1], [12], [13]. It is common for people with high implicit self-esteem to automatically associate self or self-associated objects with positive stimuli as a kind of self-positivity bias [1], [13]. Based on this understanding, a variety of measures have been employed to measure implicit self-esteem, including the Implicit Association Test (IAT, ref. [14]), semantic or affective priming paradigm [15], and Go/Nogo association task (GNAT, refs [16], [17]). Besides disassociation from explicit self-esteem [18], another robust finding about implicit self-esteem so far is its positive nature, that is, people implicitly harbor a positive view about themselves. The positive nature of implicit self-esteem consistently has been demonstrated across different measures [18] and cultures [19], ethnicities [20], and age groups [20], as well as in comparison to different social dynamics (such as others, ingroups, best friends, etc., refs [19], [20]; for a review, see ref. [21]). Given this consistency, interesting questions from the perspective of cognitive neuroscience are how and when this ubiquitous positive implicit self-view manifests on or is reflected in neural activity. We will address this issue using event-related potentials (ERPs) that offer high temporal resolution and enable us to investigate dynamic time courses for neural information processes [22].

Based on our conceptualization of implicit self-esteem, we can see that implicit self-esteem involves processing affective valence or evaluative information of the implicit self. Studies from the cognitive neural perspective about the self are numerous (for reviews, see refs [2], [23], [24]). Early studies have mainly focused on neural representations of the cognitive self and found that cortical midline structures, such as medial prefrontal and posterior cingulate cortices, are relevant to self-referential processing [25], [26]. Recently, research has examined evaluative self-processing [27]–[29] and implicit self-processing [30], [31]. When people explicitly performed self-evaluations, functional magnetic resonance imaging (fMRI) research discovered that the ventral anterior cingulate cortex [28], medial prefrontal cortex, and orbitofrontal cortex were all involved [27]; and ERP research found that the self-positivity bias manifested on the N400 component in the time course measured between 450 ms and 600 ms after stimulus onset [29]. When people processed self-relevant information implicitly, fMRI research found similar regions were involved in processing self-information explicitly, such as the medial prefrontal cortex, posterior cingulate/precuneus, etc. [30]. Similarly, ERP research found that implicit self-processing occurred during a perceptual analysis stage as indicated in P200 [31], [32]. In addition, new research showed that self-esteem modulates neural responses when people receive social feedback [33], [34] or when people complete special tasks such as self-evaluation [35], implicit self-processing [36], math problems [37], and visual probes [38]. A recent study revealed that multi-modal frontostriatal connectivity underlies individual differences in self-esteem [39]. To date, however, neural studies that examined implicit self-processing rarely have involved affective nature or evaluation of the self [30], [31]. Neural studies that involved affective nature and evaluative processes of the self are mostly based on self-report rather than implicit measures [27], [28]. Consequently, neural studies that examine implicit self-esteem or self-evaluations tapped by implicit measures are still rare.

Among the various measures for implicit self-esteem, we opted for GNAT in the present study [17]. GNAT is a classical measure of implicit attitude, or the strength of association between a target and *good* vs. *bad* attributes. A self-esteem GNAT involves at least two blocks [16], [40]. In one block (*self* + *good* condition), participants respond to *self* and *good* stimuli (Go), but ignore *others* and *bad* stimuli (Nogo) (“Press if a *self* word or *good* word”); in the other block (*self* + *bad* condition), participants respond to *self* and *bad* stimuli (Go), but ignore *others* and *good* stimuli (Nogo) (“Press if a *self* word or *bad* word”). If individuals respond faster and/or make fewer errors in the *self* + *good* condition than in the *self* + *bad* condition, they exhibit implicit self-positivity.

In the area of cognitive neuroscience, the Go/Nogo paradigm has been widely used to study neural mechanisms behind response inhibition, which is typically indicated by the N200 component in ERP [41]–[45]. As an index of response inhibition, the augmented N200, in particular the fronto-central N200 [46], frequently has been observed in Nogo responses in comparison with Go responses [47], [48]. The onset of N200 indicates the time at which the information to determine Go/Nogo decision comes available [48]–[50]. In an attitude GNAT, both Go and Nogo responses involve congruent or incongruent pairs of stimuli. When a category pair is incongruent (e.g. *self* + *bad*), participants suppress initially activated response tendencies before making a Go/Nogo decision. This inhibition may interfere and set back a Go/Nogo decision, leading to a delayed Nogo N200 component. That is, the attitude would modulate the Nogo N200 negativity [47], [48].

A recent electrophysiological study has examined neural activity underlying the attitude GNAT, specifically, a GNAT measuring an implicit attitude toward *fruit* vs. *bugs* [47]. Consistent with previous findings, results revealed an augmented N200 negativity in Nogo responses compared with Go responses. Moreover, the onset latency of this N200 negativity or the N200 difference wave obtained from Nogo minus Go were delayed in an incongruent condition (Press if a “fruit” word or a “bad” word) by a priori *fruit-good* association. Based on the timing of the N200 difference wave, the authors inferred that automatic attitude information (i.e., *fruit-good association*) was available about 250 ms after the onset of the stimuli, which is notably earlier than what was previously derived from behavioral data (i.e., between 600–700 ms). Similarly, in another study, examining the timing of Nogo negativity, van der Lugt et al. [51] found that the implicit attitude toward young vs. old people was activated between 170–230 ms after the onset of the target stimuli. These studies suggested that N200 negativity across Nogo and Go trials is useful in studying the timing of automatic attitude activations.

Based on these studies [47], [51], we focused on the N200 in the present study, elicited by *self*-stimulus in a self-esteem GNAT. We aimed to examine how and when the self-positivity association would manifest on the N200 component. We hypothesized that Nogo responses to *self* would elicit a larger (or more negative) N200 relative to Go responses. Moreover, this self-positivity association would delay the onset of N200 in difference waves obtained from Nogo minus Go in the *self* + *bad* condition compared with the *self* + *good* condition. For purposes of comparison and control, we also used a second GNAT to measure implicit attitude toward *others*. This *others* GNAT used identical stimuli to the self-esteem GNAT, but instead asked participants to “press if an *others* word or a *good* word” in an *others + good* condition and “press if an *others* word or a *bad* word” in an *others + bad* condition. Previous studies showed that a person's attitude toward *others* is neutral or negative [52], [53]. Hence, for the *others* GNAT, the latency changes in the N200 difference wave from Nogo minus Go would be trivial or in the opposite direction across *others* + *good* and *others* + *bad* conditions. As a result, interaction between a target (*self* vs. *others*) and valence (*good* vs. *bad*) would be observed. At a behavioral level, we considered reaction time to *self* and *others* in Go trials and expected a similar interaction between the target (*self* vs. *others*) and valence (*good* vs. *bad*). In particular, we believed participants would respond faster to *self* in the *self* + *good* condition than to *self* in the *self* + *bad* condition. However, the pattern in the *others* GNAT would expect not to hold.

## Method

### Ethics statement

The Local Ethics Committee at the Institute of Psychology, Chinese Academy of Sciences approved the experimental protocol. All participants gave their informed written consent prior to the experiment.

### Participants

Nineteen college students (7 women, mean age 23, all right-handed) participated in this study. Each was paid CNY50 for the compensation of their time. None of them had a demonstrated history of neurological or psychiatric disorders. All possessed normal or corrected-to-normal vision. Data from four participants (three men) were not included in the ERP analysis due to technical problems during EEG data recordings. As a result, the final sample consisted of fifteen participants (6 women; age: *Mean* = 22.9 years, *SD* = 2.7 years).

### Materials

We selected 170 Chinese words as stimuli: 5 *self* words including self, me, myself, I, and mine; 5 *others* words being he/she, him/her, his/her, other (“??”, meaning other in Chinese) and other (“??”, also meaning other); as well as 80 *good* or *positive* attribute words and 80 *bad* or *negative* attribute words. Most attributes were selected from the Chinese version of the Anderson Word List [54]; the remaining attributes were selected from a Chinese word list developed by a previous study that examined Chinese (implicit and explicit) self-esteem. The visual/perceptual complexity of *self* and *others* words indexed by the number of strokes was comparable, *Mean* = 10.80, 9.60, *SD* = 4.38, 3.85, respectively, *t*_(8)_ = 0.46, *p* = .66.

### Procedure

Two GNATs included four blocks: *self + good*, *self + bad*, *others + good*, and *others + bad*, measuring automatic attitudes toward the self (*self + good* and *self + bad*) and others (*others + good* and *others + bad*), respectively. In each block, four identical categories of stimuli were presented, one at a time. Different blocks, however, required participants to respond to different pairs of stimuli (signal) but ignore other stimuli (noise). For example, in the *self + good* block, participants were instructed to press the space bar if a stimulus conveyed *self* words or *good* words (e.g., *me* and *delight*), but to do nothing if a stimulus was *other* words or *bad* words (e.g., *he* and *bragging*). The sequence of four blocks was counterbalanced across participants. Before each block, pilot trials were run to enable participants to become familiar with the task.

Each block included 320 trials. For each trial, the stimulus was randomly selected from four categories of stimuli, with equal numbers of stimuli from each category. The target stimuli *self* and *others* were repeatedly used because each of them only comprised five variations. The attribute words, *good* and *bad*, were presented without repetition. The ratio of signal to noise was 1:1 in each block.

**Figure 1:**
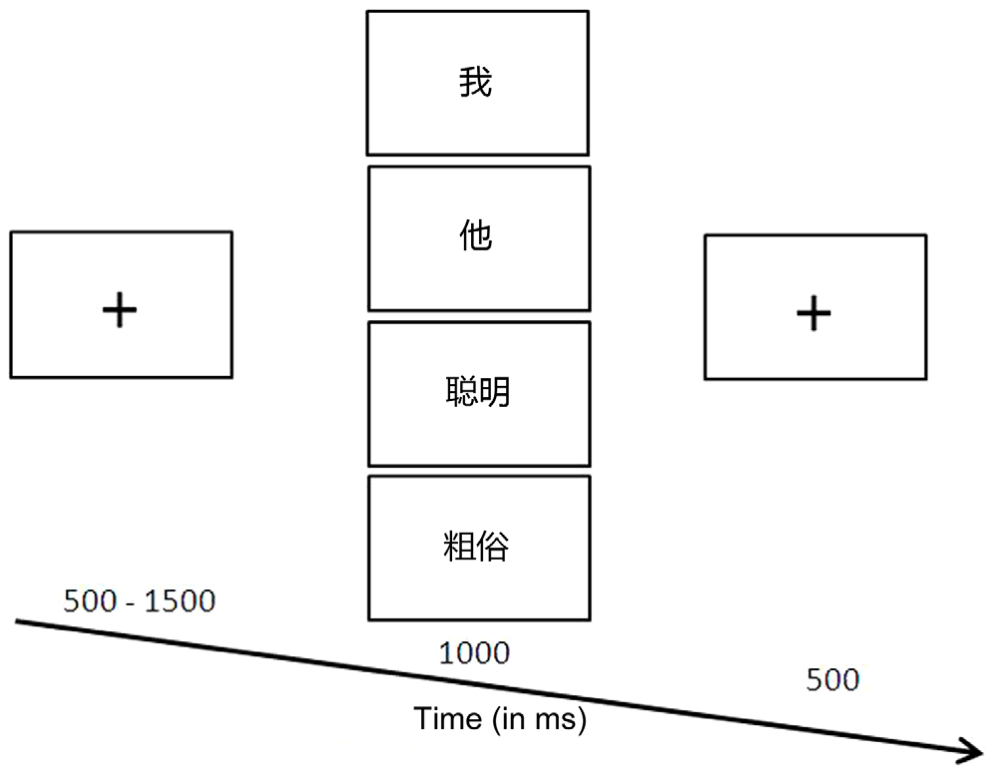
Illustration of the experimental procedure. The stimulus was randomly selected from four categories of stimuli (in this illustration, “?” = *self*, “?” = *others*, “??” = *bright*, “??” = *crude*).

Figure 1 shows the sequence of stimuli presentations. At the start of each trial, a fixation cross (‘+’) was centrally presented with a randomized duration between 500 and 1500 ms. After that, the stimulus was presented in the center of the screen for 1000 ms, and participants were required to press the spacebar if the stimulus belonged to signal categories or otherwise register no response. Next, the second fixation was presented for 500 ms. Finally, another trial started anew with the appearance of another fixation.

### EEG data recording and analysis

Continuous electroencephalogram (EEG) was recorded from 64 scalp sites using Ag/AgCl electrodes mounted on an elastic cap (NeuroScan Inc., Herndon, VA, USA) with an online reference to the right mastoid and off-line algebraic re-reference to the average of left and right mastoids. The vertical electrooculogram (VEOG) and horizontal electrooculogram (HEOG) were recorded from two pairs of electrodes, with one placed above and below the left eye and another one 10 mm from the outer canthi of each eye. The impedances of all electrodes were maintained below 5 kΩ. EEG and EOG were filtered using a 0.05–100 Hz bandpass filter and sampled at 500 Hz.

During the offline analysis, EEG data were digitally filtered with a 35 Hz low-pass filter. A regression procedure implemented in the Neuroscan software removed ocular artifacts from filtered EEG data [48]. The onset of stimuli was set as the zero time point, and continuous EEG data were epoched into segments of 1000 ms long, including a 200 ms pre-stimulus baseline. Epochs with artifacts due to eye blinks, amplifier clippings, and bursts of electromyographic (EMG) activity exceeding ±100 µV were excluded. ERPs for different word categories and two types of response (*Go* or *Nogo*) were obtained by averaging corresponding epochs from four blocks separately. Data from epochs with incorrect responses and extremely slow responses (i.e., reaction times larger than three standard deviations from mean) were not included during averaging. Finally, eight ERPs for four word categories and two response types were created.

To quantify the Nogo N200 negativity, we measured the mean amplitude of N200 within 250–450 ms at six anterior sites: F3, Fz, F4, FC3, FCz, and FC4, and then conducted a five-way ANOVA with target (*self* vs. *others*), valence (*good* vs. *bad*), response type (*Go* vs. *Nogo*), Anterior-Central (F vs. FC) and Laterality (left vs. midline vs. right) as within-subject variables.

To examine the influence of implicit self-esteem on N200 negativity in the Nogo condition relative to the Go condition, the difference wave from Nogo minus Go was computed first for each participant. Then the onset latencies of N200 negativity or N200 in difference waves were assessed through the jackknife method, which is resistant to individual noise [56], [57]. To carry this out, we obtained a new Nogo N200 difference waveform for each participant by averaging the N200 difference waveforms from all other participants in each block. We then measured the total area under the new Nogo N200 difference wave in the time window of 200–500 ms. The onset latency was defined as the time point where a pre-specified fraction (20% in this case) of the total area was reached. Therefore, for each participant, the combination of the jackknife method and fractional area latency measure produced the onset latency of the N200 difference wave. These onset latencies were entered into a 2 (*self* vs. *others*) ×2 (*good* vs. *bad*) ANOVA. The Greenhouse–Geisser correction was used to compensate for sphericity violations. The amended results were then reported in line with previous studies [55], [56]. That is, the statistical results (*F*-values and *t*-values) were corrected using the formulas: *F*_C_ = *F*/(N−1)^2^, and *t*_C_ = *t*/(N−1), where N denotes the number of observations in each condition.

## Results

### Behavioral Results

To examine whether implicit self-esteem manifested on behavioral data, we performed an ANOVA on reaction time to *self* and *other* words in Go trials with the target (*self* vs. *others*) and valence (*good* vs. *bad*) as two within-subject factors. Participants responded faster to target stimuli paired with *good* words (*Mean* = 502 ms, *SD* = 54 ms) than to those paired with *bad* words (*Mean* = 518 ms, *SD* = 50 ms), *F*_(1, 14)_ = 6.51, *p* = .023. But there was no significant difference in the response speed to *self* (*Mean* = 505 ms, *SD* = 56 ms) and *others* (*Mean* = 514 ms, *SD* = 47 ms), *F*_(1, 14)_ = 1.58, *p* = .23. As expected, the interaction was significant, *F*_(1, 14)_ = 31.88, *p*<.001. Additional simple effect tests showed that participants responded faster to *self* words in the *self + good* (*Mean* = 481 ms, *SD* = 53 ms) condition than in the *self* + *bad* condition (*Mean* = 530 ms, *SD* = 50 ms), *t*_(14)_ = −5.12, *p*<.001. In contrast, they responded faster to *other* words in the *others* + *bad* condition (*Mean* = 523 ms, *SD* = 48 ms) than in the *others* + *good* condition (*Mean* = 506 ms, *SD* = 47 ms), *t_(14)_* = 2.39, *p*<.05. These findings suggest that people implicitly have a positive attitude toward themselves, which is consistent with established implicit self-positivity [21].

### ERP Results

#### Mean amplitude of N200

We first checked if the classical Nogo vs. Go N200 negativity existed. Given individual difference in the appearance of N200, we considered a relatively large time window that was between 250 and 450 ms. The mean N200 amplitudes were measured and submitted to an ANOVA with target (*self* vs. *others*), valence (*good* vs. *bad*), response-type (Go vs. Nogo), Anterior-Central (F vs. FC), and Laterality (left vs. middle vs. right) as the within-subject variables. Consistent with past research [41], [47], [51], the target, regardless of *self* or *others*, in Nogo trials (*Mean* = 1.87 uV) elicited a larger N200 than in Go trials (*Mean* = 4.11 uV), *F*_(1, 14)_ = 34.32, *p*<.001, suggesting the suppression of motor responses in Nogo trials. No other significant effect was found, all *F*s<4.6 and all *p*s>.05.

#### Onset latency of Nogo N200

**Figure 2:**
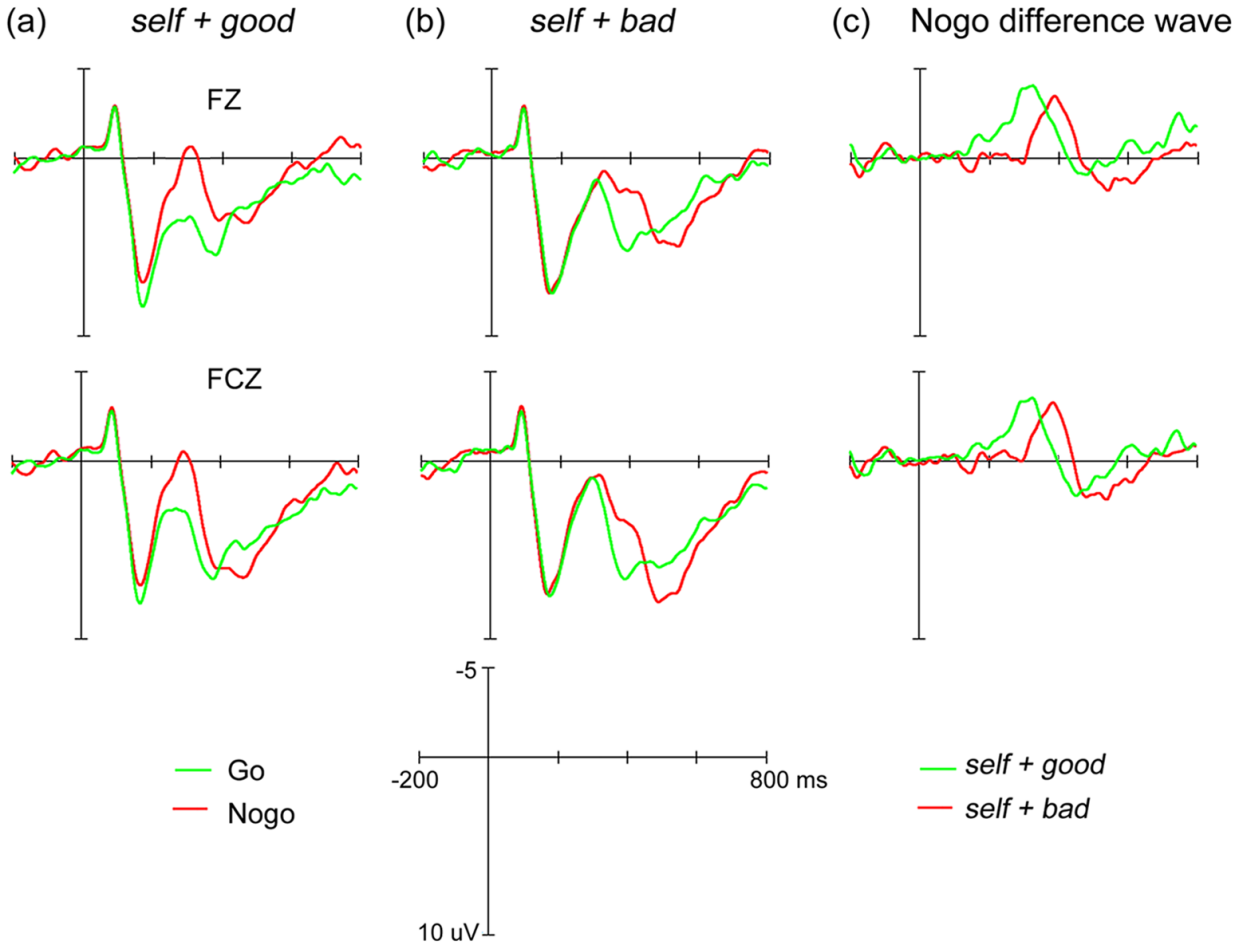
Grand-averaged ERPs for *self* words.

**Figure 3:**
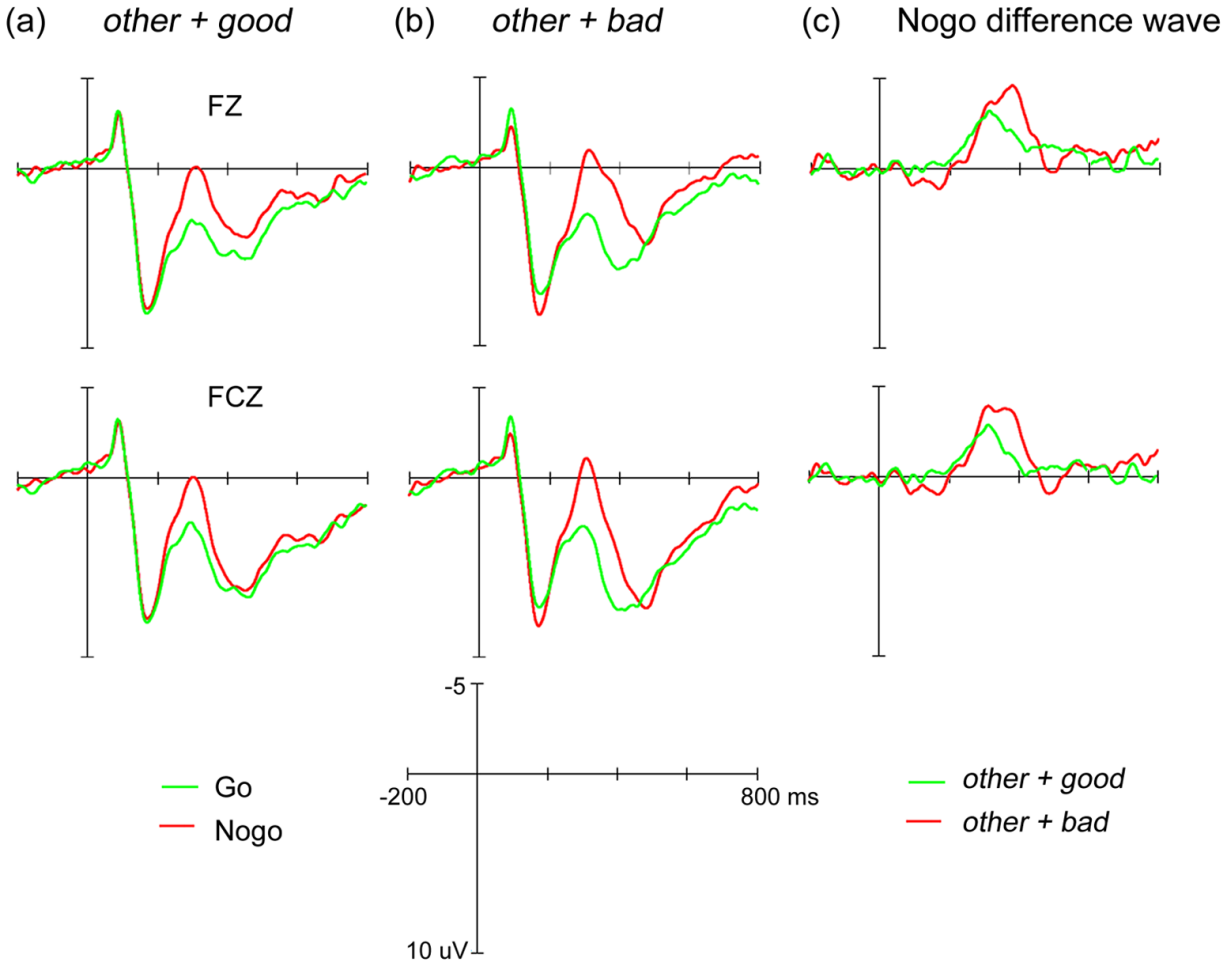
Grand-averaged ERPs for *others* words.

The N200 components elicited by *self* and *other* words with Go responses and Nogo responses as well as their difference waves computed from Nogo minus Go waves are displayed in Figures 2 and 3, respectively. Based on previous suggestions (for a review, see ref [46]), in each of these Figures, we presented the ERP waveforms from only two locations: Fz and FCz. Visual inspection suggests that the Nogo N200 negativity in the *self + bad* condition appears later than in the *self + good* condition. No visible difference, however, exists between the *others + bad* and *others + good* conditions. To examine the timing of the Nogo N200 negativity, the onset latencies of the N200 difference waveforms across all four conditions were obtained through the jackknife approach using the 20% criterion and then submitted into a 2 (*self* vs. *others*) ×2 (*good* vs. *bad*) ANOVA.

Results demonstrated that augmented Nogo N200 negativity clocked an earlier onset time (about 275 ms) when targets were paired with *good* attributes than when they were paired with *bad* attributes (about 327 ms), *F*_(1, 14)_ = 1572.52, *F_C_*_(1, 14)_ = 8.02, *p*<.05. As expected, the interaction was significant, *F*_(1, 14)_ = 1291.80, *F_C_*_(1, 14)_ = 6.59, *p*<.05. Further simple effect analysis showed that the N200 difference appeared later in the *self + bad* condition (347 ms) than in the *self + good* condition (271 ms), *t* = −46.68, *t_C_* = −3.33, *p*<.01, whereas no significant difference in Nogo N200 negativity onset latency was recorded between the *others + good* (278 ms) and *others + bad* (306 ms) conditions, *t* = −16.64, *t_C_* = −1.19, *p*>.1. Since the onset time indicated the point in time at which the evaluative information for the attitude target was available, the current findings suggested that after seeing the *self* relevant stimuli, self-evaluative information would be activated and available in less than 271 ms, which is much earlier than behavioral data have been suggested (about 600 ms).

## Discussion

Humans implicitly possess a positive view about themselves. We examined how and when this positive implicit self-esteem influences or manifests on brain activity using high temporal resolution ERPs. We measured implicit self-esteem using GNAT and focused on the ERP component of N200 that had been widely studied under the GNAT paradigm. Our behavioral data replicated findings in previous studies [16], [40]. Participants responded faster to s*elf* stimuli paired with *good* words than those paired with *bad* words, whereas they responded slower to *others* stimuli paired with *good* words than those paired with *bad* words, indicating the positive nature of implicit self-esteem. At the neural level, classic Nogo N200 negativity resulted. More important, the *self-positivity* association delayed Nogo N200 negativity in the *self* + *bad* condition as compared with the *self* + *good* condition, suggesting the manifestion of implicit self-esteem on brain activity. Notably, two early ERP components elicited by *self* and *others* words, P100 and N100, were comparable, *F*s<2.87, and *p*s>0.11, suggesting that the influences of the stimuli featured are negligible and that our main findings are not confused by irrelevant factors.

Behavioral evidence for the positivity of implicit self-esteem in humans is sizeable. Greenwald and Banaji [1] have summarized three kinds of evidence: experimental implicit self-esteem (e.g., mere ownership effect); naturally mediated implicit self-esteem (e.g., liking for name-letters); and second-order implicit self-esteem (e.g., self-positivity in judgment). The subsequent large body of research about implicit self-esteem provides further behavioral evidence for the positive nature of implicit self-esteem (for a review, see ref [21]). Our research adds to existing literature by providing novel neural evidence for the positivity of implicit self-esteem. Typical N200 negativity associated with Nogo responses relative to Go responses is delayed in the *self* + *bad* condition compared with the *self* + *good* condition, suggesting that activated *self-association* is positive and, moreover, modulates brain activity. This result is consistent with previous findings that show automatic attitudes modulate the onset of N200 [47], [51]. People may wonder why humans possess such a positive self-bias. In examining the biological mechanism of another human positive bias, i.e., optimism bias, Sharot and her colleagues suggested that selective registration of more positive than negative self-information is an important cause [58]–[60]. Similar mechanisms may also explain positive implicit self-esteem because selective updating of self-information may have made self-positive associations more accessible than self-negative associations. These have led to more efficient processing of self-information in *self + good* condition than in *self + bad* condition, which, of course, represents a new direction for future study.

Our study also sheds light on the timing in processing implicit self-associative information. Behavioral responses can only suggest the endpoint of information processing and provide little information about the process of self-associative information. Since the onset of the N200 indicates the time at which attitude information is available [47], [51], with the help of the ERP technique, we have identified that implicit self-positivity is activated and available in less than 270 ms. This time is notably earlier than the time indicated in behavioral response data, i.e., between 600 and 700 ms. Since processing speed is a core indicator of the automaticity of cognitive processes, particularly in the case of implicit social cognition [61], these findings undoubtedly provide convincing evidence about automatic nature of implicit self-esteem. Using a similar methodology, previous studies showed that attitude information about fruit versus bugs and old people versus young people is activated and available in less than 250 ms after the onset of corresponding stimuli [47], [51]. This activiation time is somewhat earlier than what we observed for implicit self-esteem, i.e., 270 ms. The difference in activation time might reflect distinct natures of attitude targets (e.g., fruit vs. self) and their representative stimuli in the two studies, or alternatively, might simply suggest random variation. Nevertheless, these findings in toto stand as general evidence for the automaticity of implicit attitude.

In addition, we used the *others* GNAT as a control in this study. Behavioral data replicated past findings [52], whereby humans possess a different implicit attitude toward *others* than toward *selves*. Neural responses in the *others* GNAT, i.e., in *others* + *good* and *others* + *bad* blocks, however, exhibit a different pattern. The onset times of Nogo N200 negativity or N200 difference waveform do not differ across conditions. Attitudes toward the *others* factor similarly do not influence neural activity as implicit self-esteem does. These findings provide neural evidence that people's implicit attitudes toward others are distinct from attitudes toward themselves. More importantly, this study also rules out the possibility that the difference between *self* + *good* and *self* + *bad* conditions is due to paired attribute valence (*good* vs. *bad*). Thus, utilizing the *others* GNAT as a control, we are more confident that implicit self-esteem (or implicit *self-positivity* bias), rather than the valence of paired categories, modulates the latency of Nogo N200 negativity. People might wonder why an implicit negative bias against *others* manifests in a behavioral index, but not in a neural index. Research has shown that behavioral outcomes of implicit measures, such as GNAT, are the result of cumulative output from many processes, including both automatic and controlled processes [63]. It is possible that some controlled processes have influenced behavioral outputs. Humans have a basic need for a positive self [64], [65] and downward social comparison serves as a common way for people to enhance self-positivity [66]. It has also been suggested that automatic social comparison can influence implicit self-evaluation [67]. Therefore, the negative bias manifesting in a behavioral index might be caused by the tendency of enhancing self by looking down on others, which should be further studied in the future.

Recently, implicit self-esteem, particularly as elucidated by the Implicit Assocation Test [14] and Name Letter Preference [12], has been challenged due to its dubious predictive power [21]. In light of this concern, one may question the significance of examining neural substrates of implicit self-esteem. Low predictive capability, however, is not necessarily equated with low validity of a measure or a construct [62], particularly for implicit measures of social cognition [63]. The establishment of predictive validity is usually based on presumed nomological principles in terms of behavioral criteria, which may be misleading due to the limitations of the nomological network as well as the correlational nature of evidence. Hence, the low predictive power of implicit self-esteem may not suggest its low validity and subseqent inefficacy, but rather, highlights the importance of looking into the nature of implicit self-esteem using alternative methodologies. We believe the cognitive neuroscience approach constitutes a promising new way given its exquisite utility in revealing the cognitive neural basis of a construct or a psychological process. In this sense, our work represents an innovative attempt from the perspective of cognitive neuroscience. We demonstrate that implicit self-esteem is reflected in neural activity by modulating the onset time of the N200 difference wave (Nogo minus Go). We hope that more studies from a cognitive neuroscience perspective will appear in the near future and will help to clarify further the nature of implicit self-esteem.

In conclusion, we demonstrated the electrophysiological signature of implicit self-esteem and revealed relevant timing features for the processing of early self-associative information. These findings provide novel evidence for the positivity and automaticity of implicit self-esteem. Future studies may examine how implicit self-esteem reflects on other neural activities.

## Supporting Information

Text S1 **Results of the N200 elicited by** ***good*** **and** ***bad*** **words.**
(DOCX)
