# Supplementary material for: Neural Manifestations of Implicit Self-Esteem: An ERP Study
Source: PLoS One. 2014 Jul 9;9(7):e101837. doi: 10.1371/journal.pone.0101837 (PMC4090159; doi:10.1371/journal.pone.0101837)
Supplement: Text S1 — Results of the N200 elicited by good and bad words. (DOCX) [file pone.0101837.s001.docx]

**Text S1:** Results of the N200 elicited by *good* and *bad* words.

Identical analyses were carried out on the N200 elicited by *good* and *bad* words. Results showed that the attribute words elicited a larger N200 in Nogo trials (*Mean* = 4.23 uV) than Go trials (*Mean* = 5.79 uV), regardless of the valence of words, *F* _(1, 14)_ = 26.23, *p* < .001, which replicated the classic Nogo N200 negativity. No other effects on the N200 were significant, all *F*s < 1.99 and all *p*s > .18. As to the onset time of the N200 in difference waveforms from Nogo versus Go, no significant effects were noted, all *F*s < 64, all *F_C_*s < 0.323, and all *p*s > .05.
